# Vector-borne parasites in dogs from Ukraine translocated to Poland following Russian invasion in 2022

**DOI:** 10.1186/s13071-023-06042-2

**Published:** 2023-11-21

**Authors:** Anna Bajer, Mustafa Alsarraf, Mariia Topolnytska, Katarzyna Tołkacz, Dorota Dwużnik-Szarek, Anna Rodo

**Affiliations:** 1 Department of Eco-Epidemiology of Parasitic Diseases, Institute of Developmental Biology and Biomedical Sciences, Faculty of Biology, University of Warsaw, 1 Miecznikowa Street, 02-096 Warsaw, Poland; 2 Institute of Biochemistry and Biophysics, Polish Academy of Sciences, Pawińskiego 5A, 02-106 Warsaw, Poland; 3 Department of Pathology and Veterinary Diagnostics, Faculty of Veterinary Medicine, Warsaw University of Life Sciences - SGGW, 159C Nowoursynowska Street, 02-766 Warsaw, Poland

**Keywords:** *Dirofilaria*, *Hepatozoon*, Ukraine, Poland, Pets, Cat

## Abstract

**Introduction:**

Since 24 February 2022, the day the Russian aggression against Ukraine began, millions of refugees and thousands of pets crossed the Polish-Ukrainian border. Additionally, an unknown number of shelter and stray dogs and cats were rescued and translocated to Poland by private persons and non-profit organizations. The aim of the present study was to examine rescued dogs and cats for presence of canine vector-borne parasites to determine the role of armed conflict in spreading these parasites.

**Methods:**

In July 2022 blood samples were collected from two animal shelters in central Poland hosting dogs and cats rescued from Ukraine. Animals were imported from various regions of Ukraine, including eastern and southeastern Ukraine (military conflict area). Fifty-three dogs (51 from two shelters and two owned ones) and one shelter cat were examined by molecular methods (PCR and sequencing) for the presence of *Babesia/Theileria* spp., *Dirofilaria* spp. and *Hepatozoon* spp. DNA.

**Results:**

We detected *Dirofilaria immitis*, a parasite species non-endemic in Poland, in two dogs translocated from Ukraine (2/53 = 3.8% [95% CI 0.5–13.0%]). One dog had a history of previous heartworm infection. High prevalence of *Hepatozoon canis* (51% [95% CI 36.8–64.9%]) was noted among translocated dogs. Prevalence of *Dirofilaria repens* in Ukrainian dogs (18.9% [95% CI 9.4–32.0%) was similar to prevalence in dogs from central Poland (12%). Co-infection of *D. repens* and *D. immitis* was found in two dogs and six dogs were co-infected by *D. repens* and *H. canis*. *Hepatozoon canis* infection was also identified in an imported cat.

**Conclusion:**

We confirmed that this military conflict has facilitated the spread of canine vector-borne parasites, including zoonotic species.

**Graphical Abstract:**

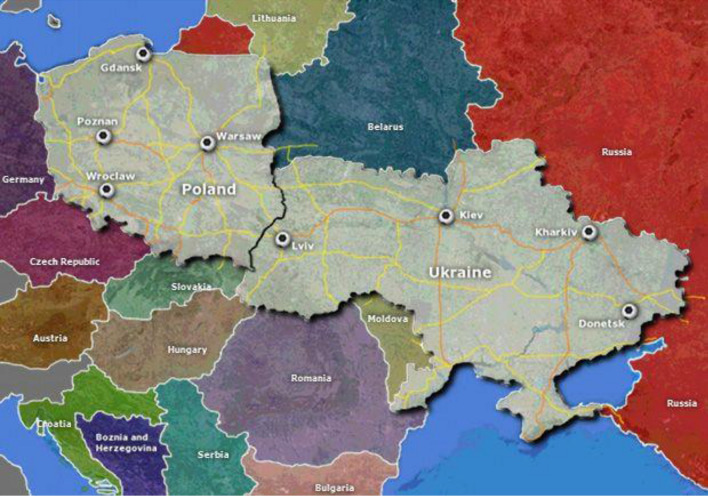

**Supplementary Information:**

The online version contains supplementary material available at 10.1186/s13071-023-06042-2.

## Short communication

Since 24 February 2022, the day the Russian aggression against Ukraine began, about one-third of Ukraine’s 41 million citizens have been forced to leave their homes. This is the biggest displacement crisis in the world in recent time. According to United Nations (UN) data, more than 6.6 million refugees from Ukraine have been registered in Europe so far, most of them in Poland, Russia and Germany [1].

Since the beginning of the war up to December 2022, the Polish-Ukrainian border has been crossed by 8.7 million refugees from Ukraine (data by Polish Border Guard). The number of refugees from Ukraine in 2022 in Poland was estimated at 1.1 million, mostly women and children [2]. Many of these families have fled to Poland with their pets. To facilitate the crossing of the Polish-Ukrainian border with pets, the Chief Veterinary Officer released a simplified temporary procedure for dealing with animals translocated for non-commercial purposes and accompanying refugees entering the European Union (EU) from Ukraine through the Polish border [3]. This procedure was designed to control rabies and focused on ensuring effective vaccination against rabies in dogs, cats and ferrets and tracking the identification (via microchipping) and intended location of the animal. Due to massive traffic at the border, no other preventive measures have been implemented to control other infectious diseases, including parasitic diseases.

Massive support for Ukrainian refugees from Polish citizens [2] has been coupled with the support for animals living in conflict areas. This was necessary as thousands of dogs and cats were abandoned in Ukraine at the beginning of the war, which increased the number of strays. In war conditions animal shelters in Ukraine have not been able to provide enough care for their animals. Many people were devastated by the case of Borodyanka shelter near Kiev, where only 150 dogs out of 485 survived being locked in their kennels without food and water for 3 weeks while the shelter was inaccessible (area of conflict) [4]. Thus, many stray or abandoned dogs and cats were rescued and translocated to Poland by non-governmental organizations (NGOs) and private volunteers [5].

It is hard to evaluate how many dogs and cats, stray and owned, from Ukraine have been translocated to Poland since the beginning of the Russian military operation. About 6700 importation documents from the Chief Veterinary Inspectorate and 11,500 importation of ‘changes in procedure’ documents have been registered [3]. Probably more than 10,000 animals have crossed the Polish-Ukrainian border, and some were then placed in animal shelters.

The aim of this study was to estimate the prevalence of important vector-borne parasite infections in dogs imported from Ukraine to assess the impact of the military conflict/refugee crisis on the spread of infectious diseases. We focused on two mosquito-borne pathogens, *Dirofilaria repens* and *D. immitis*, which are zoonotic and have high potential to be introduced in non-endemic areas by infected dogs and cats [6–8]. *Dirofilaria immitis* is not endemic in Poland yet, and *D. repens* is newly endemic with rising numbers of canine and human cases [8]. We also examined these animals for infection with important tick-borne pathogens, *Babesia/Theileria* spp. and *Hepatozoon canis*.

## Methods

### Ethical issue

Since the study was carried out on blood samples provided voluntarily for diagnostic purposes by veterinary services of animal shelters, no ethical approval or license was required for our study (as per Resolution on the Protection of Animals Used for Scientific or Educational Purposes, 15 January 2015 [9]). The owner of two dogs involved in this study was informed about the aims of the study and provided oral consent and contact information to obtain the results of testing. The directors and veterinary services of shelters were informed about the results of testing of the dogs under their care.

Blood samples from 53 dogs (51 strays, two owned) and one cat from two shelters providing care for dogs translocated from Ukraine in central Poland (Mazovia region) were collected into EDTA tubes in July 2022. The total number of dogs imported from Ukraine was about two times higher in these shelters; however, samples were taken only from dogs (and the cat) that were deemed suitable (e.g. not fearful or aggressive) for sample collection. Two owned dogs (border collies) belonged to an Ukrainian refugee working at one of the shelters. Additionally, due to limited number of available tests, 11 of the oldest dogs (> 10 years) with the highest risk of heartworm infection were tested for heartworm antigen at the shelter with SNAP 4Dx, IDEXX, Maine, USA.

Genomic DNA was extracted from the samples using a commercial kit [10]. Detection of *Dirofilaria* spp. was done by amplification and sequencing of two genetic markers, 12S rDNA and COI gene, as previously described [10, 11]. *Babesia/Theileria* spp. and *Hepatozoon* spp. infections were diagnosed by nested PCR and sequencing [12, 13].

The evolutionary history of *H. canis* was inferred by using the maximum likelihood method and Tamura three-parameter model [14]. The analysis involved 36 nucleotide sequences. There were a total of 392 positions in the final dataset. Evolutionary analyses were conducted in MEGA X [15].

The evolutionary history of *D. repens* was inferred by using the maximum likelihood method and Tamura three-parameter model [14]. A *D. immitis* sequence (OQ727018) obtained from GenBank was used as an outsource group.

The evolutionary history of the single *D. immitis* was inferred by using the maximum likelihood method and Tamura three-parameter model [14]. A *D. repens* sequence (KX265049) obtained from GenBank was used as an outsource group (Additional file 1: Fig. S1).

## Results

**Fig. 1:**
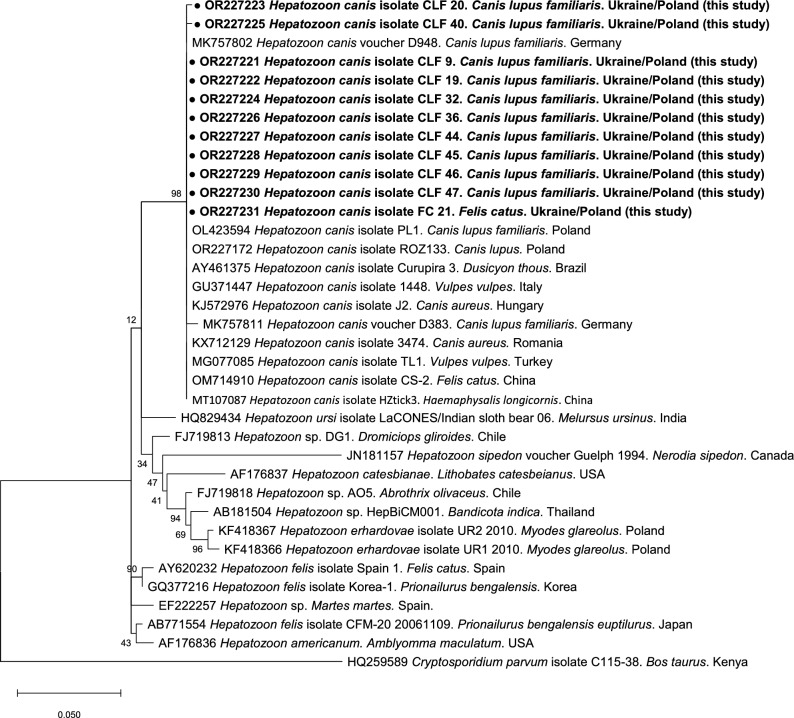
Evolutionary history of *Hepatozoon* based on the fragment of the *18S rRNA* gene was inferred by using the maximum likelihood method and Tamura three-parameter model [14]. The tree is drawn to scale, with branch lengths measured in the number of substitutions per site. This analysis involved 36 nucleotide sequences. The codon positions included were 1st + 2nd + 3rd + Noncoding. All positions containing gaps and missing data were eliminated (complete deletion option). In the final dataset, there were 392 positions. The nucleotide sequence of *Cryptosporidium parvum* was used as an outgroup. Evolutionary analyses were conducted in MEGA v. X [15]

*Hepatozoon canis* DNA was identified in 27 dogs (51%, 95% CI 36.8–64.9%) and one cat. There was minimal diversity among the obtained sequences displaying 99.4–100% identity to *H. canis* voucher D948 (MK757802) derived from a dog in Germany [18]. Representative *18S rRNA* sequences have been deposited in GenBank under accession numbers OR227221–OR227231, and phylogenetic analysis confirmed identification of *H. canis* in a cat and dogs from Ukraine (Fig. 1).

Two dogs tested positive for *D. immitis* antigen (3.8%, 95% CI 0.5–13.0%), and infection was confirmed by sequencing of PCR product in one of these dogs. Additionally, one dog had been diagnosed with *D. immitis* infection at its arrival to Poland but tested negative in the current study following treatment. The obtained COI sequence (701 bp) displayed 100% identity with the sequence of *D. immitis* isolated from a human in Thailand (MW577348). The phylogenetic analysis confirmed identification of *D. immitis* in this dog (Additional file 1: Fig. S1). The sequence was deposited in GenBank under accession number OQ726945.

**Fig. 2:**
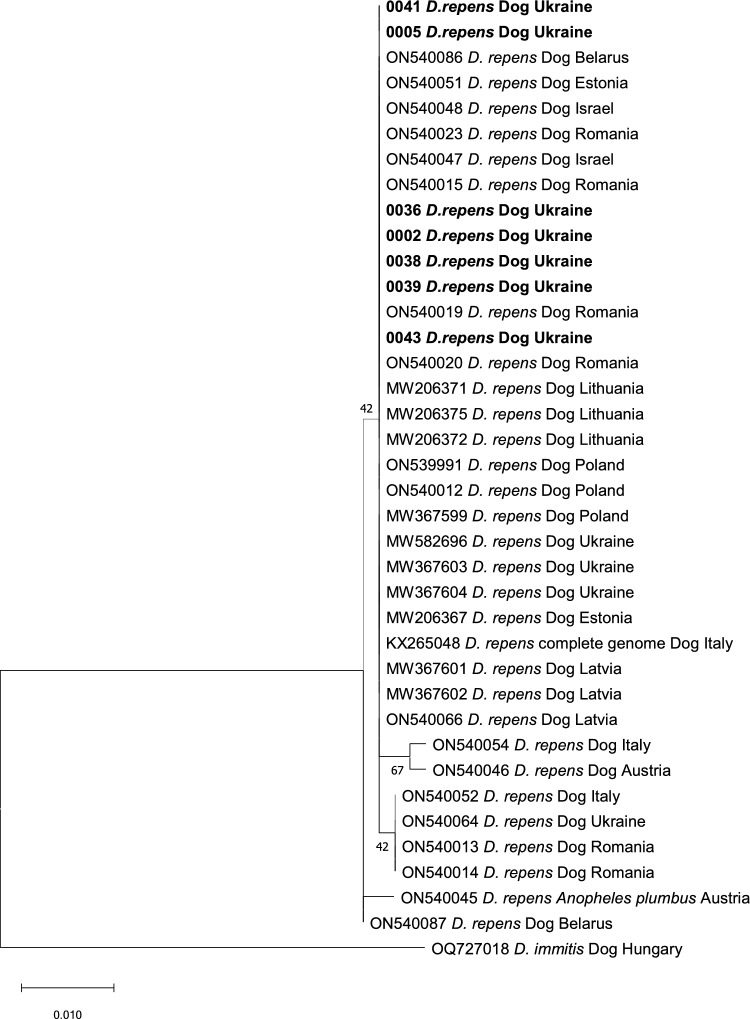
Evolutionary history of *Dirofilaria repens* was inferred by using the maximum likelihood method and Tamura three-parameter model for the COI gene. The tree with the highest log likelihood (−1047.43) is shown. The percentage of trees in which the associated taxa clustered together is shown next to the branches. Initial tree(s) for the heuristic search were obtained automatically by applying neighbor-joining and BioNJ algorithms to a matrix of pairwise distances estimated using the Tamura 3 parameter model and then selecting the topology with superior log likelihood value. The tree is drawn to scale, with branch lengths measured in the number of substitutions per site. This analysis involved 38 nucleotide sequences. Codon positions included were 1st + 2nd + 3rd + Noncoding. There was a total of 596 positions in the final dataset. Evolutionary analyses were conducted in MEGA X [15]

*Dirofilaria repens* DNA was detected in ten dogs (18.9% [95% CI 9.4–32.0%]) including two dogs positive for *D. immitis* by antigen test (SNAP 4Dx). Eight obtained sequences of COI gene fragment displayed 100% identity with the sequence of *D. repens* isolated from a human case in Croatia (KX265049) and with the numerous sequences of *D. repens* from dogs from Europe. All *D. repens* COI sequences from Ukraine grouped with other *D. repens* sequences on the phylogenetic tree (Fig. 2). Four obtained 12S rDNA sequences displayed 100% identity with the sequences of *D. repens* isolated from dogs in Poland (KX265080, KX265088).

Co-infection of *D. repens* and *D. immitis* was found in two elderly dogs. One of these dogs was also infected with *H. canis* (triple infection). In six dogs (11.3% [95% CI 4.3–23.0%]), *H. canis* and *D. repens* co-infections were confirmed by PCR and sequencing. DNA of *Babesia/Theileria* spp. was not detected in tested animals.

## Discussion

Compared to dogs examined previously in Mazovia region in central Poland [10, 16], we have detected new incursions of *H. canis* and *D. immitis* infection via dogs imported from Ukraine and hosted in two animal shelters in this region of the country.

Although *H. canis* infections were recently reported in dogs in countries neighbouring Poland (i.e. the Czech Republic, Slovakia and Germany) [17–19], this parasite is rare in Poland. The first two cases were found in 2020 and 2022 in southern and central Poland, respectively [20]. This is likely because of the absence of tick vector (*Rhipicephalus sanguineus* complex). In contrast, *H. canis* was the most common parasite identified in dogs translocated from Ukraine (prevalence > 50%) and was also found in one examined cat. Interestingly, in a previous study of 23 owned dogs sampled in the spring of 2011 in Kiev, only one dog (prevalence 4%) was PCR-positive for *H. canis* [20]; thus, our study may indicate a rapid increase in prevalence of *H. canis* in dogs in Ukraine over the last 10 years. Another explanation may be a higher prevalence in dogs from other regions of Ukraine or higher exposure in stray than in owned dogs.

High prevalence of *H. canis* in animals from Ukraine is more comparable to the prevalence in free-living carnivores in Poland (35% in grey wolves and 46% in red foxes; [21, 22]) and this may reflect easy spread of this parasite in stray/rural animals which may resort to scavenging [23, 24]. The impact of this high prevalence of *H. canis* on animal health in Poland appears to be of less importance because these shelter animals seem to be dead-end hosts for the parasite in the absence of a suitable tick vector.

Prevalence of *D. repens* was almost 20% in translocated dogs. *Dirofilaria repens* has recently become endemic in Poland [8]. The first autochthonous *D. repens* infections of dogs were reported in Poland in Mazovia between 2009 and 2011 [25–28]. The first cases of human dirofilariasis, including likely autochthonous cases, were described between 2007 and 2009 [29–31]. Between 2007 and 2011, a total of 18 *D. repens* infections were detected in humans in Poland [32]. In subsequent epidemiological studies in dogs, high prevalence of infection (25–57%) was reported in central Poland, especially in southern Mazovia [16, 33, 34]. DNA of *D. repens* was also recently found in red foxes, grey wolves and European badger from the same area of Poland [35]. In the most recent studies, the prevalence of *D. repens* in dogs from Poland was about 12% (for years 2017, 2019, 2020) [8, 10, 36].

In 2019, we examined 155 owned dogs from Western Ukraine and found a relatively low prevalence of *D. repens* (3.9%) [10]. However, much higher prevalence was reported in owned dogs from Kiev area, where *D. repens* infection was identified in six dogs (6/23 = 26.1% [10.2–48.4%]; two were PCR positive, and in four *D. repens* was identified by proxy in engorged ticks) [20]. Interestingly, there were similarities in haplotype structure/distribution among Polish and Ukrainian *D. repens* sequences, suggesting regional circulation of haplotypes [11]. Thus, high prevalence noted among dogs translocated from Ukraine is comparable to the current prevalence in Poland and supports known endemicity in this region of Europe.

In contrast, *D. immitis* is still not endemic in Poland [8, 10] so translocation of infected dogs can result with introduction of this parasite to the country. In 2012, the first, likely autochthonous, case of *D. immitis* infection was recognised in a dog in Gdynia, northern Poland [37]. However, no additional cases have been reported to date, imported or autochthonous [8, 10, 36]. *Dirofilaria immitis* is endemic in Ukraine, and although the actual prevalence in dogs is not determined/reported, one *D. immitis*-infected dog was identified among 23 owned dogs from Kiev (4% in [20]). A number of human cases have also been reported [38, 39]. In our study, we have confirmed one *D. immitis* infection by both molecular methods (PCR and sequencing) and antigen presence, second infection was only detected by antigen presence, and one dog was reported with a known history of heartworm infection (currently negative following treatment). Thus, overall prevalence of *D. immitis* in this small group of dogs was rather high (5.7%, 95% CI 1.2–15.7%) and comparable to the prevalence reported in other endemic countries [7].

As *D. repens* is already endemic in Poland, suitable climatic and vectoral conditions probably exist also for transmission of *D. immitis*, and translocated infected dogs may serve as the source of infections for mosquitoes and then local dogs and humans.

The main limitation of our study is the relatively small number of animals studied, including only one cat. The sample size is small compared to the likely large number of translocated pets accompanying Ukrainian refugees in Poland. Another limitation is the lack of data on the animals included in the sample, especially on their previous location in Ukraine, health status or ownership (stray or owned dogs). These data were mostly lost in the chaos caused by the armed conflict and the refugee crisis.

## Conclusions

Our study revealed that military conflict resulting in massive translocation of animals may facilitate the spread of infectious diseases.

### Supplementary Information

**Additional file 1: Figure S1.** The evolutionary history of *Dirofilaria immitis* was inferred by using the maximum likelihood method and Tamura three-parameter model of the COI gene. The tree with the highest log likelihood (– 1115.50) is shown. The percentage of trees in which the associated taxa clustered together is shown next to the branches. Initial tree(s) for the heuristic search were obtained automatically by applying neighbor-joining and BioNJ algorithms to a matrix of pairwise distances estimated using the Tamura three-parameter model and then selecting the topology with superior log likelihood value. The tree is drawn to scale, with branch lengths measured in the number of substitutions per site. This analysis involved 48 nucleotide sequences. Codon positions included were 1st + 2nd + 3rd + Noncoding. There were a total of 649 positions in the final dataset. Evolutionary analyses were conducted in MEGA X [15].

## Data Availability

All relevant data are included in the article. Representative *18S rRNA* sequences of *H. canis* were deposited in GenBank under accession numbers OR227221–OR227231. Obtained *D. immitis* sequence was deposited in GenBank database under accession number OQ726945.

## Abbreviations

EU: European Union
NGO: Non-Governmental Organisation
PCR: Polymerase chain reaction
rRNA: Ribosomal ribonucleic acid
UN: United Nations
