# Supplementary figures and images for: Vector-borne parasites in dogs from Ukraine translocated to Poland following Russian invasion in 2022
Source: Parasit Vectors. 2023 Nov 21;16:430. doi: 10.1186/s13071-023-06042-2 (PMC10664295; doi:10.1186/s13071-023-06042-2)

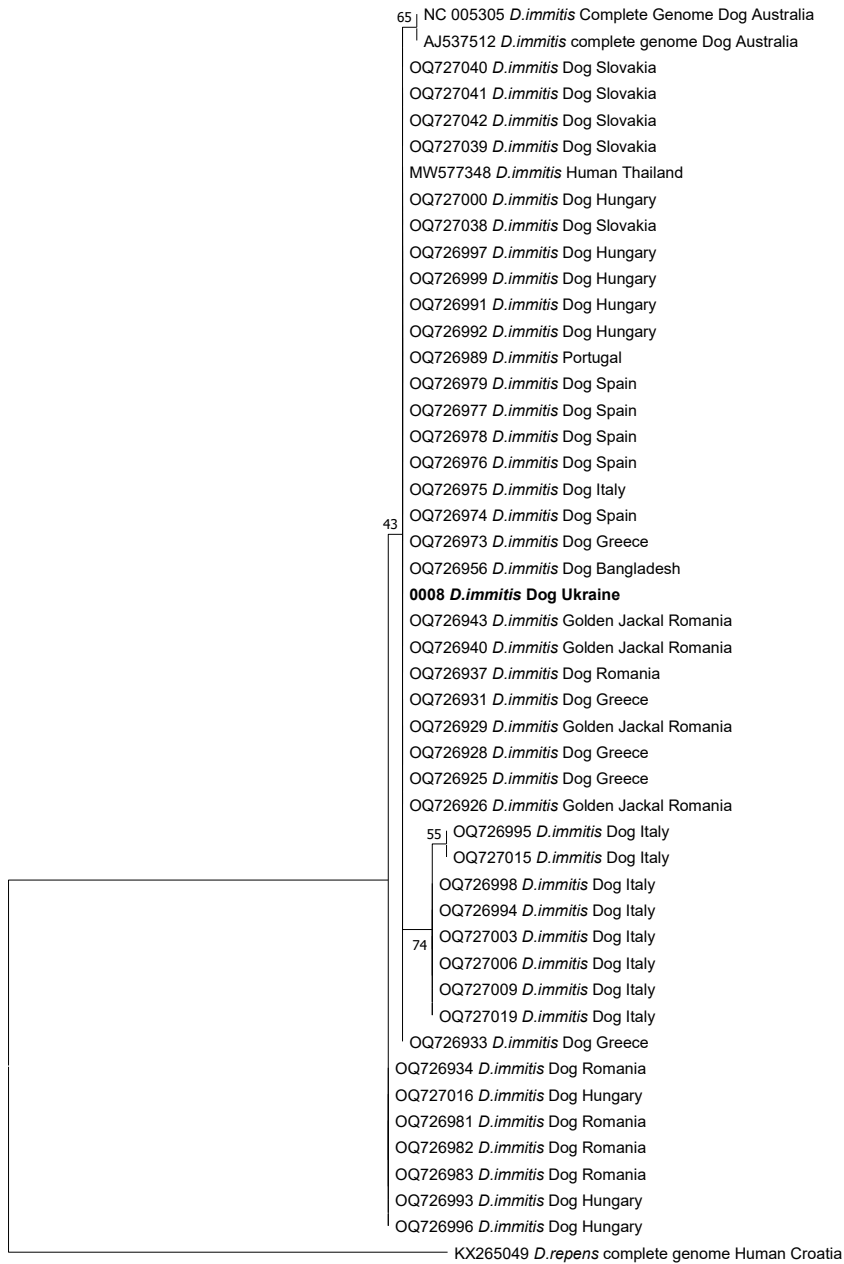

Supplement: Supplementary file 1 — Additional file 1: Figure S1. The evolutionary history of Dirofilaria immitis was inferred by using the maximum likelihood method and Tamura three-parameter model of the COI gene. The tree with the highest log likelihood (– 1115.50) is shown. The percentage of trees in which the associated taxa clustered together is shown next to the branches. Initial tree(s) for the heuristic search were obtained automatically by applying neighbor-joining and BioNJ algorithms to a matrix of pairwise distances estimated using the Tamura three-parameter model and then selecting the topology with superior log likelihood value. The tree is drawn to scale, with branch lengths measured in the number of substitutions per site. This analysis involved 48 nucleotide sequences. Codon positions included were 1st + 2nd + 3rd + Noncoding. There were a total of 649 positions in the final dataset. Evolutionary analyses were conducted in MEGA X [15]. [file 13071_2023_6042_MOESM1_ESM.pdf]
